# The use of artificial intelligence models for interpretation and triage of clinical referral letters to acute and specialist care pathways: Protocol for a systematic review

**DOI:** 10.1371/journal.pone.0355476

**Published:** 2026-08-06

**Authors:** James Foley, Enda Hession, Etimbuk Umana, Fiona Boland

**Affiliations:** 1 Data Science Centre, School of Population Health, Royal College of Surgeons in Ireland (RCSI), University of Medicine and Health Sciences, Dublin, Ireland; 2 Department of Emergency Medicine, University Hospital Galway, Galway, Ireland; 3 School of Medicine, University of Galway, Ireland; 4 Department of Emergency Medicine, Children’s Health Ireland at Crumlin, Dublin, Ireland; 5 Department of Emergency Medicine, Mater Misericordiae University Hospital, Dublin, Ireland; University of Kwazulu-Natal, SOUTH AFRICA

## Abstract

**Background:**

Clinical referral letters direct patients into acute, urgent, and specialist care pathways, but their triage is typically manual, heterogeneous, and resource intensive, contributing to waiting-list pressure and potential patient harm. Artificial intelligence (AI) methods, including natural language processing, machine learning, and large language models, offer potential to automate or augment referral triage, yet the performance, safety, and implementation characteristics of AI applied to referral documentation have not been systematically synthesised. A February 2026 PROSPERO search identified no completed or ongoing systematic reviews addressing this question.

**Objectives:**

To evaluate the prioritisation performance of AI models used to triage clinical referral documentation for acute and specialist care pathways, and to map how this emerging field defines and evaluates AI-assisted referral triage, including model types, reference standards, validation practices, safety outcomes, and implementation-related outcomes.

**Methods:**

This protocol is reported in accordance with PRISMA-P. Systematic searches will be undertaken in MEDLINE, EMBASE, Web of Science, Scopus, CINAHL, and the Cochrane Central Register of Controlled Trials, covering January 2016 to the 5 May 2026, with no language restrictions. Eligible studies will include diagnostic accuracy, model development and validation, and comparative studies that apply an AI model to referral documentation and report at least one triage-performance outcome. Two reviewers will independently screen, extract data, and assess risk of bias using PROBAST-AI as appropriate. A structured narrative synthesis and evidence map using the SWiM reporting guideline will be the primary synthesis output, stratified by AI model class and clinical pathway. Meta-analysis will be treated as conditional and exploratory, and will be undertaken only where studies report comparable outcomes using a similar model class, clinical setting, reference standard, outcome definition, and threshold structure.

**Expected outcomes and significance:**

This review will produce the first systematic synthesis and evidence map of AI-assisted referral triage across acute and specialist care pathways, characterising how the field defines and evaluates this task as well as what is currently known about prioritisation performance. Findings will inform clinicians, service planners, and policymakers considering AI adoption in referral workflows, and will identify methodological priorities for future research.

**Systematic review registration:** PROSPERO CRD420251244654.

## Introduction

### The problem

Referral letters and clinical documentation are central to directing patients into acute healthcare. Across health systems, patients are referred from primary care, emergency settings, community services, or other clinical teams into acute, elective, urgent, or tertiary-level services. These referrals rely on clinical documentation, including referral letters, clinical notes, clinical summaries, or electronic referral forms, which all vary considerably in quality, structure, and completeness [1–3]. The triage of these referrals determines urgency, waiting times, and access to care, and is a major contributor to service delays across acute and specialist pathways [4–6].

The triage and prioritisation of referrals is frequently slow, inconsistent, and resource intensive [7]. In many health systems, this process is performed manually by clinicians or experienced nursing staff, who must integrate heterogeneous and often incomplete information to assign clinical priority [8–10]. This can be challenging, time-consuming, and cause frustration to the individuals triaging the referrals, and subsequent delays to the patient for whom the referral was sent. Under and over-triage of referral letters has been reported, and the subjectivity of the triage disposition often depends on the quality of the information in the letter, and the experience of the individual triaging [10].

As populations increase, the demand on health systems increases concurrently, and the volume of referrals that must be triaged becomes increasingly difficult with these challenges [7]. Waiting lists in healthcare are a focus of intent for health governing bodies internationally, and current triage systems to reduce these lists have no adopted universal framework, nor are they built to manage a rapidly rising referral burden. The triage and prioritisation of referral documentation across acute and specialist care represents an analogous challenge: a process that is slow, inconsistent, and reliant on manual clinical review of heterogenous documentation.

### AI as a potential solution

Artificial intelligence (AI), in particular machine learning (ML), deep learning (DL), and natural language processing (NLP) have significant and rapidly expanding potential to support clinical decision-making. NLP systems can analyse free-text clinical documentation to extract clinically relevant features and classify patient urgency, if the system has been trained to a high standard [11,12]. ML models have demonstrated the ability to predict triage acuity, disposition, and clinical deterioration from structured patient data and early clinical observations in emergency settings while large language models (LLMs) can generate clinical reasoning based on the acquisition of extensive clinical knowledge, which represents a further development for AI-assisted triage from unstructured referral text [12–14]. Collectively, these approaches could offer a scalable, consistent, efficient and potentially safer adjunct or alternative to purely manual referral triage, with the capacity to reduce delays and improve prioritisation accuracy at scale.

### Evidence gap

Despite this promise, the performance, safety, and clinical implementation characteristics of AI models for the triage of referral documentation have not been systematically synthesised. A search of PROSPERO conducted in February 2026 identified no completed or ongoing systematic reviews specifically evaluating AI models for interpretation and triage of clinical referral letters across acute and specialist care pathways. Existing reviews have addressed adjacent topics including AI for triage using physiological data and structured EHR inputs, AI for diagnostic support in specific clinical domains, and AI for the interpretation of diagnostic imaging and clinical notes in specific specialties but none has evaluated the specific intersection of free-text or semi-structured referral documentation and AI-assisted triage across the range of acute and specialist care pathways [15,16]. This gap limits the ability of clinicians, health service planners, and researchers to make evidence-informed decisions about the adoption of AI for referral triage.

### Objectives

#### Primary objective.

To evaluate the prioritisation performance of AI models used to triage clinical referral documentation, including referral letters, clinical notes and electronic referral forms for acute and specialist care pathways, and to map how this emerging field defines and evaluates AI-assisted referral triage.

Prioritisation performance will be assessed as agreement with each study’s defined reference standard and interpreted in the context of the study’s clinical pathway, reference standard and level of urgency, rather than a transferable construct.

#### Secondary objectives.

1. To map the evidence base for AI-assisted referral triage, including the types of referral documentation processed, the clinical pathways addressed, the reference standards adopted, and the reporting of calibration, internal validation, and external validation.
2. To identify and describe the types of AI models (including machine learning, deep learning, and natural language processing) developed for referral triage, including their data sources, methods of development and validation, and the clinical areas in which they have been applied.
3. To assess safety-related outcomes, with particular attention to under-triage rates, over-triage rates, and any reported adverse clinical outcomes associated with AI-based referral triage.
4. To examine implementation-related outcomes, including efficiency, feasibility, interpretability, and acceptability of AI models used in referral triage contexts.
5. To identify methodological gaps in the current evidence base relevant to the real-world adoption of AI for referral triage, and to highlight priorities for future research.

## Methods

This protocol is reported in accordance with the Preferred Reporting Items for Systematic Review and Meta-Analysis Protocols (PRISMA-P) guidelines [17]. The completed systematic review will be conducted and reported in accordance with the PRISMA guidelines [18–20]. A comprehensive systematic search will be undertaken across relevant databases. All identified records will be imported into a reference management system, and duplicates will be removed.

### Eligibility criteria

All studies that examine the use of an AI tool for triage of referral documentation will be considered against the inclusion and exclusion criteria outlined in Table 1.

**Table 1:** Inclusion criteria.

Population	Patients referred for assessment in acute, urgent, specialist, or tertiary care services, where a referral letter or clinical documentation is used to determine priority or urgency. Examples include General Practice (GP) referrals, eReferrals, and consultation requests sent to Emergency Departments, Acute Medical or Surgical Units, Outpatient Departments, or equivalent services. All patient populations; adult, paediatric, or mixed will be eligible, provided that the referral is intended for review by an acute or specialist service.
Intervention	AI models of any type applied to referral documentation (free-text or structured) to support triage or prioritisation in an acute or specialist care pathway. This includes, but is not limited to: 1. Natural language processing (NLP) and large language models (LLMs) processing referral letter text. 2. Machine learning (ML) classifiers applied to structured referral fields or EHR data accompanying the referral. 3. Deep learning models processing clinical documentation. The AI system must produce or inform a triage category, urgency classification, acuity score, or prioritisation decision as its primary output.
Comparator	Clinical decision-making by a healthcare professional (for example, consensus expert clinician or healthcare provider decision regarding the appropriate triage or prioritisation determination based on the referral letter). Usual care referral processes. Studies evaluating AI triage against a clearly defined clinical reference standard even in the absence of a direct human comparator will be included if the reference standard is clinically validated and explicitly described.
Setting	Any acute or specialist care pathway internationally, including Emergency Departments, Acute Medical Units, Surgical Assessment Units, Outpatient Departments, and equivalent services in any country or healthcare system.
Outcomes	Studies must report at least one outcome related to triage performance, including but not limited to: • Accuracy or agreement with a reference standard • Sensitivity, specificity, or predictive values • Measures of triage appropriateness or prioritisation accuracy • Time to triage decision or efficiency metrics
Study Design	Eligible study designs will include: • Diagnostic accuracy studies • Retrospective or prospective cohort studies • Model development and/or validation studies (including internal and external validation) • Randomised or non-randomised comparative studies evaluating AI-assisted triage Studies must include a quantitative evaluation of AI performance against a comparator or reference standard.

#### Exclusion criteria.

Studies will be excluded if they,

- Do not involve referrals to acute or specialist care pathways (for example, administrative, social, or non-clinical referrals).
- Do not include a documentation component to a referral, or if the AI model was purely used for administrative purposes.
- Do not include a triage or prioritisation standard.
- Case reports, case series, editorials, opinion pieces, narrative reviews and conference abstracts without sufficient methodological detail (unless full text is available).

### Information sources and search strategy

An electronic search strategy will be performed using MEDLINE, EMBASE, Web of Science, Scopus, CINAHL, and the Cochrane Central Register of Controlled Trials. The search strategy will be broad, and focus on four conceptual blocks, using *Artificial Intelligence, Clinical Referral or Documentation Triage/Prioritisation and Acute/Specialist Care* as key MeSH terms, exploded where available. There will be no language restrictions, papers not in English will be included and translated where necessary with translation processes documented in the review. To enhance completeness, we will perform backward and forward citation chaining of all included studies and relevant reviews. To capture emerging evidence in this rapidly developing field, we will additionally search Europe PMC for relevant preprints; these will be screened against the same eligibility criteria, flagged as non-peer-reviewed, and reported separately, and will not be pooled with peer-reviewed studies in any quantitative synthesis. The review will include studies published from January 2016–5 May 2026 (the date of the search execution), reflecting the emergence of modern LLM and deep learning methods in clinical applications and is consistent with the ten-year limit specified in the PROSPERO registration. An example search strategy is shown in *Supplementary Material, Table 1* (S1 File).

### Study records

#### Data management.

Search results will be imported into Covidence, where duplicate records will be identified and removed. Screening of titles, abstracts, and full texts will be conducted using Covidence. Data extraction will be completed using a piloted standardised extraction form in Covidence or Microsoft Excel. All data will be stored on a secure, access-restricted institutional drive with regular backups.

#### Selection process.

Two authors will independently screen the results of the search strategy, first by title and abstract, and then by examination of the full articles according to the inclusion criteria. Any unresolved discrepancy between these two authors will be resolved by the third author. A PRISMA 2020 compliant flow diagram will document the full screening process, from total records identified through each database, through deduplication, screening, full-text review, and final inclusion. Reasons for exclusion at the full-text stage will be reported. Where multiple publications report on the same model, dataset or cohort, the most methodologically rigorous manuscript will be designated the primary record, with additional reports cross-referenced and treated as supplementary to avoid double counting. Successive versions of the same model (e.g., GPT 3.5 versus GPT 4.0 evaluations) will be documented and reported separately in the synthesis.

### Data extraction and data items

Two authors will extract data independently using a standardised extraction form developed within Covidence or Microsoft Excel. The form will be piloted on a random subset of three to five studies before formal extraction commences and refined as necessary. Discrepancies will be resolved through discussion, with a third reviewer consulted where consensus cannot be reached. All extraction decisions will be documented.

Missing data will be documented and reported as ‘not reported’ where applicable. Authors will be contacted for clarification where key data is missing. Analyses will be conducted using available data, and the potential impact of missing data on the findings will be considered in the interpretation of results.

The data items to be extracted, where possible, are presented in Table 2.

**Table 2:** Summary of components for the data extraction tool.

**Study Characteristics**	Author(s), year of publication, country, and clinical context Sample size, funding sources, setting of study, study design
**Population**	Patient population and relevant case-mix Referral source Care pathway or service receiving the referral
**AI Intervention**	AI model type and architecture Specific algorithm or model Model class (ML, NLP, Deep Learning, LLM) Model version and release date (where reported) Fine-tuning or adaptation methods (where reported) Input data: type and source Output data Training data source, size, and augmentation methods Validation methodology Calibration reporting Error analysis Explainability or interpretability mechanisms
**Comparator / Reference Standard**	Description of comparator Number and type of healthcare providers forming the reference standard Consensus method for reference standard deviation Whether the standard was blinded to AI output
**Primary Outcomes: Prioritisation Performance**	Sensitivity and specificity with 95% confidence intervals Positive predictive value (PPV) and negative predictive value (NPV) Area under the ROC curve (AUROC / AUC) Agreement statistics Overall percentage accuracy
**Secondary Outcomes: Safety**	Under-triage rate Over-triage rate Adverse clinical outcomes attributable to or associated with AI triage decisions Near-miss of safety incident data
**Secondary Outcomes: Implementation**	Time to triage or prioritisation decision Downstream clinical outcomes and referral pathway data Clinician acceptability and usability System feasibility and integration characteristics Cost or resource implications

### Risk of bias in individual studies

The risk of bias will be assessed independently by two reviewers. The following validated tools will be applied according to study design.

- AI Prediction Model Development and/or Validation: PROBAST-AI where applicable [21,22]
- Randomised Controlled Trials: Cochrane Risk of Bias 2 (ROB 2) [23]
- Non-randomised comparative studies: ROBINS-I (Risk of Bias in Non-Randomised Studies of Interventions) [24]
- Diagnostic accuracy studies evaluating an AI model against a clinical reference standard without explicit model development: QUADAS-2 ([25]

Where a study combines elements of multiple designs (for example, model development with concurrent prospective validation), the most appropriate tool will be selected, and the rationale documented. Disagreements in risk of bias judgements will be resolved by discussion; third-reviewer adjudication will be sought where consensus cannot be reached.

### Data synthesis

Given the anticipated clinical, methodological, and statistical heterogeneity of included studies, results will be synthesised primarily using a structured narrative approach as the primary method, supplemented by quantitative synthesis where feasible. Metrics such as accuracy, sensitivity, specificity, and AUC may not be directly comparable across studies even when reported in a similar form, because their meaning depends on the reference standard, and the clinical consequence of over- and under-triage in each pathway. Accordingly, terms such as “performance” and “agreement” will be interpreted in relation to each study’s own reference standard and pathway, not as generalisable transferable constructs.

The review therefore has two complimentary synthesis aims. The first is a structured evidence map of the field, describing the types of AI models utilised, the types of referral documentation processed, the clinical pathway, and the reference standards assessed. The second is a synthesis of prioritisation performance, agreement with reference standards, and safety and implementation outcomes, if meaningful comparison can be achieved.

For both aims, a narrative synthesis will be conducted using the SWiM (Synthesis Without Meta-Analysis) reporting guideline, with structured summaries of studies tabulated to enable comparison of the primary and secondary objectives [26]. The synthesis will be stratified by (i) AI model class (traditional machine-learning classifiers, NLP pipelines, deep learning models, and LLM-based systems), reflecting their differing validation requirements and failure modes, and (ii) clinical pathway (emergency/acute versus outpatient/elective), reflecting differences in the clinical meaning of under- and over-triage. Stratification by model class will extend beyond performance to how each class has been evaluated, including validation designs, reference standards, and reported failure modes. Meta-analysis will be treated as a conditional and exploratory output and will be performed only where a minimum of three studies report comparable outcomes using a similar AI model class, clinical setting, reference standard, outcome definition, and threshold structure. Quantitative analyses will be conducted using StataNow 19.5 SE [27]. Where diagnostic accuracy meta-analysis is appropriate, bivariate random-effects or hierarchical summary receiver operating characteristic (HSROC) models will be used, depending on the availability of sensitivity and specificity estimates and the degree of threshold variation across studies.

Where meta-analysis is not feasible, results will be described narratively with reference to the direction and magnitude of effects across studies.

### Reporting bias

Reporting bias will be assessed by considering potential publication bias and selective outcome reporting. Where sufficient studies are available (typically ≥10 and sufficiently homogeneous), funnel plots may be generated to explore potential small-study effects. Statistical tests for funnel plot asymmetry (e.g. Egger’s test) will be considered where appropriate. In addition, where study protocols or trial registry entries are available, reported outcomes will be compared against prespecified outcomes to assess selective outcome reporting. However, it is anticipated that there will be too few studies to explore funnel plots, and availability of study protocols may be limited in AI-based studies.

### Certainty of evidence

GRADE will not be applied as the review focuses on AI model development and performance rather than intervention effects on patient outcomes. Instead, methodological quality and risk of bias will be assessed as outlined above.

### Study status and timeline

This review has been prospectively registered with PROSPERO (CRD420251244654; https://www.crd.york.ac.uk/PROSPERO/view/CRD420251244654). Database searches were completed on 5 May 2026, and title/abstract screening is ongoing at the time of submission. No full-text screening, data extraction, risk-of-bias assessment, or synthesis has yet been undertaken. We anticipate the following timeline: (A) title/abstract and full-text screening will be completed by August 2026; (B) data extraction and risk-of-bias assessment will be completed by November 2026; and (C) data synthesis and final results are expected by February 2027. Any substantive amendments to the protocol after registration will be recorded in the PROSPERO record and reported transparently in the final review.

### Protocol amendments

Any amendments to this protocol after the date of PROSPERO registration and prior to the completion of the review will be documented in a dated protocol-amendment log, with each amendment recorded as follows: (1) date of amendment; (2) section of protocol affected; (3) nature of the amendment; (4) rationale for the amendment; and (5) anticipated impact of the amendment on the review findings.

Substantive amendments affecting the review question, eligibility criteria, information sources, search strategy, outcomes, risk-of-bias assessment, or synthesis approach will also be reflected in an updated PROSPERO registration, with the version history maintained transparently in the registry. Minor amendments, such as corrections to typographical errors or clarification of pre-specified procedures that do not alter the review’s methodology, will be recorded in the internal amendment log only.

All deviations from the protocol encountered during the conduct of the review will be reported in the final review manuscript, in a dedicated subsection of the Methods, in accordance with PRISMA 2020 [20].

## Discussion

This review will provide the first systematic synthesis of evidence on the use of AI models for the interpretation and triage of clinical referral documentation across acute and specialist care pathways. A central contribution of the review will be to map how this emerging field defines and evaluates AI-assisted referral triage including the reference standards used, the urgency-category structures adopted, and the reporting of calibration and external validation so that subsequent performance comparisons can be interpreted in their proper clinical and methodological context. The results will be of value to health services where referral volumes are high, and triage is performed manually by nursing or medical staff. If AI models are found to demonstrate high sensitivity and specificity for urgent case identification, then policymakers and service planners may consider incorporating AI-assisted triage into referral workflows, particularly in conjunction with a sequential or hybrid human-AI assessment. This has the potential to reduce delays to clinical review, improve triage consistency, and support safer prioritisation of high-acuity referrals.

The planned synthesis will also allow comparison across AI model types and input modalities, including NLP approaches applied to free-text letters, structured eReferral data, and hybrid inputs. If specific model architectures or input types are found to have comparable or superior performance, this may provide a basis for prioritising their development and evaluation in settings where more resource-intensive approaches are currently unavailable or impractical.

A key strength of this review is its broad scope across care pathways and settings, and its application of PROBAST-AI, the methodological standard specifically developed for AI prediction model studies. The inclusion of studies from multiple countries and service configurations will increase the generalisability of findings.

The main anticipated limitation is the absence of a standardised definition of triage across the included literature, with studies likely applying a range of triage scales and institution-specific thresholds. This will require caution in interpreting pooled accuracy estimates and may limit comparability across settings. More fundamentally, given that triage is a context-dependent clinical prioritisation judgement rather than a single stable diagnostic target, superficially similar metrics may carry different meaning across studies, allowing for the proposed meta-analysis to be treated as exploratory rather than a central expected output. A second limitation is the expected predominance of internal validation studies: where external or prospective validation is absent; findings should be interpreted conservatively with respect to generalisability. Both limitations will be addressed explicitly in risk of bias assessments and will inform recommendations for future research.

## Supporting information

S1File Supplementary material. (DOCX)

S2 File PRISMA-P 2015 checklist. (DOCX)
