# Supplementary material for: The use of artificial intelligence models for interpretation and triage of clinical referral letters to acute and specialist care pathways: Protocol for a systematic review
Source: PLoS One. 2026 Aug 6;21(8):e0355476. doi: 10.1371/journal.pone.0355476 (PMC13446580; doi:10.1371/journal.pone.0355476)
Supplement: Supplementary file 1 [file pone.0355476.s001.docx]

S1: Supplementary Material

*Table 1. Sample Search Strategy for Ovid MEDLINE*

| **#** | **Query** | **Results from 5 Mar 2026** |
| --- | --- | --- |
| 1 | (artificial intelligence or machine learning or large language* or LLM or deep learning or natural language processing or clinical decision support* or generative AI or neural network).mp. [mp=ti, bt, ab, ot, nm, hw, fx, kf, ox, px, rx, ui, an, sy, ds, on, ux, mx, cw, tx, sh, ct] | 471,955 |
| 2 | limit 1 to yr="2015 - 2026" | 427,617 |
| 3 | (referral* or consultation or correspondence or letters or GP letter or primary care referral or electronic referral or specialist referral or clinical letter).mp. [mp=ti, bt, ab, ot, nm, hw, fx, kf, ox, px, rx, ui, an, sy, ds, on, ux, mx, cw, tx, sh, ct] | 854,104 |
| 4 | 1 and 2 and 3 | 9,954 |
| 5 | (triage or clinical decision-making or risk assessment or manchester* or streaming or prioriti*).mp. [mp=ti, bt, ab, ot, nm, hw, fx, kf, ox, px, rx, ui, an, sy, ds, on, ux, mx, cw, tx, sh, ct] | 949,811 |
| 6 | 4 and 5 | 2,561 |
| 7 | (emergency* or ambulatory care or secondary care or hospital or clinic or outpatient* or health services or ED or urgent care or specialist care).mp. [mp=ti, bt, ab, ot, nm, hw, fx, kf, ox, px, rx, ui, an, sy, ds, on, ux, mx, cw, tx, sh, ct] | 4,462,953 |
| 8 | 6 and 7 | 1,712 |
