# Supplementary material for: The use of artificial intelligence models for interpretation and triage of clinical referral letters to acute and specialist care pathways: Protocol for a systematic review
Source: PLoS One. 2026 Aug 6;21(8):e0355476. doi: 10.1371/journal.pone.0355476 (PMC13446580; doi:10.1371/journal.pone.0355476)
Supplement: Supplementary file 2 [file pone.0355476.s002.docx]

**PRISMA-P 2015 Checklist**

The use of artificial intelligence models for interpretation and triage of clinical referral letters to acute and specialist care pathways: protocol for a systematic review

**Lead investigator and guarantor:** Dr James Foley

**Co-authors:** Dr Enda Hession, Dr Etimbuk Umana, Dr Fiona Boland

| **Section / Topic** | **Item** | **Checklist item** | **Status** | **Location in manuscript** | **Manuscript text / explanation** |
| --- | --- | --- | --- | --- | --- |
| **ADMINISTRATIVE INFORMATION** | | | | | |
| Title — Identification | 1a | Identify the report as a protocol of a systematic review | **Addressed** | Title page | “The use of artificial intelligence models for interpretation and triage of clinical referral letters to acute and specialist care pathways: protocol for a systematic review” — the article type is identified as a Study Protocol in the title and in the cover letter. |
| Title — Update | 1b | If the protocol is for an update of a previous systematic review, identify as such | **Not applicable** | — | This is an original protocol; it is not an update of a previously completed or published systematic review. |
| Registration | 2 | If registered, provide the name of the registry (such as PROSPERO) and registration number | **Addressed** | Abstract (Systematic review registration); Funding and Data availability statements | “PROSPERO CRD420251244654” — prospectively registered with the International Prospective Register of Systematic Reviews (PROSPERO). |
| Authors — Contact | 3a | Provide name, institutional affiliation, e-mail address of all protocol authors; provide physical mailing address of corresponding author | **Addressed** | Title page / byline | Dr James Foley (RCSI; UHG; University of Galway); Dr Enda Hession (CHI at Crumlin); Dr Etimbuk Umana (Mater Misericordiae University Hospital); Dr Fiona Boland (RCSI). Corresponding-author email and physical mailing address are provided in the submission form and on the title page. |
| Contributions | 3b | Describe contributions of protocol authors and identify the guarantor of the review | **Addressed** | Author contributions section | Contributions reported using the CRediT taxonomy. James Foley is identified as the lead investigator and guarantor of the review (Conceptualization, Methodology, Investigation, Writing – original draft, Project administration, Funding acquisition). |
| Amendments | 4 | If the protocol represents an amendment of a previously completed or published protocol, identify as such and list changes; otherwise, state plan for documenting important protocol amendments | **Addressed** | Protocol amendments section (end of Methods) | This is not an amendment of a prior protocol. A dated protocol-amendment log will record the date, affected section, nature, rationale, and anticipated impact of each amendment; substantive amendments will trigger an updated PROSPERO registration. Deviations will be reported in the final review manuscript per PRISMA 2020. |
| Support — Sources | 5a | Indicate sources of financial or other support for the review | **Addressed** | Funding statement | MD by Research candidature at the Royal College of Surgeons in Ireland. There is no external funding in place for this review; the work is self-funded by the lead investigator (JF). |
| Sponsor | 5b | Provide name for the review funder and/or sponsor | **Addressed** | Funding statement | No external funder or commercial sponsor. The review is self-funded by the lead investigator. |
| Role of sponsor or funder | 5c | Describe roles of funder(s), sponsor(s), and/or institution(s), if any, in developing the protocol | **Addressed** | Funding statement | Not applicable. As the review receives no external funding and has no sponsor, no funder or sponsor had any role in the design or development of the protocol, and none will have any role in the conduct, analysis, or reporting of the completed review. |
| **INTRODUCTION** | | | | | |
| Rationale | 6 | Describe the rationale for the review in the context of what is already known | **Addressed** | Introduction — The problem; AI as a potential solution; Evidence gap | The Introduction sets out the burden of manual referral triage and waiting-list pressure, summarises AI methods with potential to automate or augment triage (NLP, ML, DL, LLMs), and documents the evidence gap. A February 2026 PROSPERO search identified no completed or ongoing systematic reviews on this question. |
| Objectives | 7 | Provide an explicit statement of the question(s) the review will address with reference to participants, interventions, comparators, and outcomes (PICO) | **Addressed** | Objectives (Primary and Secondary); Table 1 (Eligibility criteria) | Primary objective is explicitly stated. PICO elements are operationalised in Table 1: Population (patients referred to acute/urgent/specialist/tertiary services); Intervention (AI models applied to referral documentation); Comparator (healthcare professional decision or clinically validated reference standard); Outcomes (prioritisation performance interpreted against each study’s own reference standard, safety, implementation). Five secondary objectives address mapping of the evidence base (reference standards, model classes, validation practices), model characterisation, safety, implementation, and methodological gaps. |
| **METHODS** | | | | | |
| Eligibility criteria | 8 | Specify the study characteristics (such as PICO, study design, setting, time frame) and report characteristics (such as years considered, language, publication status) to be used as criteria for eligibility for the review | **Addressed** | Eligibility criteria; Table 1 (Inclusion Criteria); Exclusion criteria list; Information sources and search strategy | Table 1 specifies Population, Intervention, Comparator, Setting, Outcomes, and Study Design criteria. Exclusion criteria are listed separately. Time frame: January 2016 to 5 May 2026 (date of search execution). No language restrictions (non-English papers translated). Publication-status handling for case reports, editorials, and conference abstracts without sufficient methodological detail is specified under Exclusion criteria; preprints and other non-peer-reviewed sources are excluded to ensure a consistent baseline of editorial and methodological scrutiny. |
| Information sources | 9 | Describe all intended information sources (such as electronic databases, contact with study authors, trial registers or other grey literature sources) with planned dates of coverage | **Addressed** | Information sources and search strategy | Databases: MEDLINE, EMBASE, Web of Science, Scopus, CINAHL, and the Cochrane Central Register of Controlled Trials. Coverage: January 2016 to 5 May 2026 (date of search execution). Supplementary sources: forward and backward citation chaining of included studies and key reviews via Scopus, and searches of trial and model registries (ClinicalTrials.gov, ISRCTN) to identify ongoing or unpublished evaluations. Authors will be contacted for clarification where key data is missing (Data extraction section). |
| Search strategy | 10 | Present draft of search strategy to be used for at least one electronic database, including planned limits, such that it could be repeated | **Addressed** | Table 2 (Sample Search Strategy for Ovid MEDLINE) | Full eight-line Ovid MEDLINE search strategy presented in Table 2, with results count as of 5 March 2026 (final set: 1,712). Four conceptual blocks: AI terms; referral/documentation terms; triage/prioritisation terms; acute/specialist-care-setting terms. Year limit “2015 – 2026” applied at line 2. |
| Study records — Data management | 11a | Describe the mechanism(s) that will be used to manage records and data throughout the review | **Addressed** | Study records — Data management | Search results imported into Covidence; deduplication, title/abstract screening, and full-text screening performed in Covidence. Data extraction in Covidence or Microsoft Excel. Data stored on a secure, access-restricted institutional drive with regular backups. |
| Selection process | 11b | State the process that will be used for selecting studies (such as two independent reviewers) through each phase of the review | **Addressed** | Study records — Selection process | Two reviewers will independently screen titles/abstracts and then full texts. Disagreements resolved by a third reviewer. A PRISMA 2020–compliant flow diagram will document records identified, deduplicated, screened, full-text reviewed, and finally included, with reasons for full-text exclusion reported. |
| Data collection process | 11c | Describe planned method of extracting data from reports | **Addressed** | Data extraction and data items | Two reviewers will extract data independently using a standardised extraction form developed within Covidence. Form piloted on a random subset of three to five studies before formal extraction. Discrepancies resolved by discussion with third-reviewer adjudication. Authors contacted for clarification where key data are missing. |
| Data items | 12 | List and define all variables for which data will be sought, any pre-planned data assumptions and simplifications | **Addressed** | Table 3 (Summary of components for the data extraction tool) | Table 3 lists extraction variables across seven domains: Study Characteristics; Population; AI Intervention (model type and class, algorithm, architecture, input/output data, training data, validation, calibration, explainability, reported failure modes); Comparator / Reference Standard; Primary Outcomes (prioritisation performance and agreement with each study’s reference standard); Secondary Outcomes (Safety); Secondary Outcomes (Implementation). Missing data to be reported as ‘not reported’. |
| Outcomes and prioritization | 13 | List and define all outcomes for which data will be sought, including prioritization of main and additional outcomes, with rationale | **Addressed** | Objectives; Table 1 (Outcomes row); Table 3 | Primary outcomes: prioritisation performance, defined as agreement with each study’s own reference standard and interpreted in its clinical and methodological context (sensitivity, specificity with 95% CIs; PPV/NPV; AUROC; agreement statistics; overall accuracy, where reported). Secondary outcomes: safety (under-triage, over-triage, adverse clinical outcomes, near-miss data) and implementation (time to decision, downstream pathway data, clinician acceptability, feasibility, cost). Prioritisation reflects clinical primacy of performance followed by safety and implementation. |
| Risk of bias in individual studies | 14 | Describe anticipated methods for assessing risk of bias of individual studies | **Addressed** | Risk of bias in individual studies | Independent assessment by two reviewers at study level using design-matched tools: PROBAST-AI for AI prediction model development/validation studies; Cochrane RoB 2 for RCTs; ROBINS-I for non-randomised comparative studies; and QUADAS-2 for diagnostic accuracy studies evaluating an AI model against a clinical reference standard without explicit model development. Where study design is mixed, the most appropriate tool is selected and the rationale documented. Disagreements resolved by discussion with third-reviewer adjudication. Risk-of-bias findings will inform the narrative synthesis. |
| Data synthesis | 15a | Describe criteria under which study data will be quantitatively synthesised | **Addressed** | Data synthesis | The primary synthesis output is a structured narrative synthesis and evidence map (SWiM), stratified by AI model class and clinical pathway, addressing both the mapping of the evidence base and prioritisation performance. Meta-analysis is treated as conditional and exploratory: it will be performed only where a minimum of three studies report comparable outcomes using a similar AI model class, clinical setting, reference standard, outcome definition, and threshold structure. |
|  | 15b | If data are appropriate for quantitative synthesis, describe planned summary measures, methods of handling data and methods of combining data from studies, including any planned exploration of consistency | **Addressed** | Data synthesis | Quantitative analyses will be conducted in StataNow 19.5 SE. Summary measures reflect diagnostic-accuracy reporting (sensitivity, specificity with 95% CIs; AUROC). Where diagnostic-accuracy data are pooled, bivariate random-effects models will be used, and hierarchical summary receiver operating characteristic (HSROC) models will be applied where thresholds differ between studies. Reporting of between-study consistency and statistical heterogeneity will be undertaken where pooling is feasible; where pooling is not feasible, results will be described narratively with reference to direction and magnitude of effects across studies. |
|  | 15c | Describe any proposed additional analyses (such as sensitivity or subgroup analyses, meta-regression) | **Addressed** | Data synthesis; Objectives (Secondary) | Stratification by AI model class (traditional ML classifiers, NLP pipelines, deep learning, LLM-based systems) and by clinical pathway (emergency/acute vs outpatient/elective) is a central organising principle of the synthesis, extending beyond performance to how each class is evaluated (validation designs, reference standards, reported failure modes). Comparisons across input modalities (free-text, structured eReferral, hybrid) and subgroup/sensitivity analyses by clinical setting and validation approach (internal vs external) will be conducted where the number of included studies permits. |
|  | 15d | If quantitative synthesis is not appropriate, describe the type of summary planned | **Addressed** | Data synthesis | A structured narrative synthesis using the SWiM (Synthesis Without Meta-Analysis) reporting guideline is the primary synthesis method, with structured tabulated study summaries to enable comparison across the primary and secondary objectives. |
| Meta-bias(es) | 16 | Specify any planned assessment of meta-bias(es) (such as publication bias across studies, selective reporting within studies) | **Addressed** | Reporting bias | Publication bias: funnel plots and Egger’s test where ≥ 10 sufficiently homogeneous studies are available. Selective outcome reporting: comparison of reported outcomes against pre-specified outcomes in available study protocols or trial registry entries. Limitations acknowledged given the expected small study count and limited protocol availability in AI-based studies. |
| Confidence in cumulative evidence | 17 | Describe how the strength of the body of evidence will be assessed (such as GRADE) | **Addressed** | Certainty of Evidence | GRADE will not be applied, as the review focuses on AI model development and prioritisation performance rather than intervention effects on patient outcomes. Instead, the strength of the body of evidence will be characterised through design-matched risk-of-bias assessment (PROBAST-AI, RoB 2, ROBINS-I, QUADAS-2) and structured appraisal of methodological quality, calibration, and internal versus external validation, as outlined above. |
