# Supplementary material for: The use of artificial intelligence models for interpretation and triage of clinical referral letters to acute and specialist care pathways: Protocol for a systematic review
Source: PLoS One. 2026 Aug 6;21(8):e0355476. doi: 10.1371/journal.pone.0355476 (PMC13446580; doi:10.1371/journal.pone.0355476)
Supplement: Supplementary file 3 [file pone.0355476.s003.docx]

**The use of artificial intelligence models for interpretation and triage of clinical referral letters to acute and specialist care pathways: protocol for a systematic review**

This topic, titled above, is quite relevant and important in primary health care, as it affects patients' waiting times to be seen at specialist referral centers and/or for other acute clinical care.

The sections are well written and in accordance with the journal's published guidelines and requirements for publishing the study protocol, as required by PLOS ONE. Each heading is accompanied by an adequate description that clarifies the steps required for the study protocol submission guidelines.

The study protocol has implications; hence, if published, the detailed protocol can be used to review the findings and advantages, enabling replication of the research in other contexts when relevant.

‘Systematic reviews aim to provide a comprehensive, unbiased synthesis of many relevant studies in a single document using rigorous and transparent methods’ which is achieved through the study protocol

**Introduction**

‘This section is well written and makes a case for introducing different models, such as AI, to tackle a common problem in the referral system. This can fast-track patient triage, enabling them to be attended to according to their priority and providing quicker, more appropriate, and optimal care. Each topic is well developed, with a clear paragraph structure and creates an argument. The aim and objectives in this study protocol are clearly stated.’

**Study Materials and Methods**

The guidelines require that they should include the aim, design, and setting of the study, the sample size, inclusion, and exclusion criteria, the characteristics of participants and how the sample will be selected, and, where applicable, randomization and blinding and description of how materials will be selected for the study

A true description of all processes, interventions, comparisons, what outcomes will be measured as primary and secondary outcomes, data management plans and a detailed description of safety considerations, bias management, and statistical analyses planned are all explained in the study protocol

In this study protocol, the researchers clearly describe how the planned systematic review will be conducted using rigorous and transparent methods.

The guidelines also require a clear explanation of the study aim, design, and setting; sample size; inclusion and exclusion criteria; participant characteristics; sample selection procedures; and, where applicable, randomization, blinding, and the process used to select studies. These components are well explained in the protocol.

All of the following are explicitly detailed in the study protocol: a true description of all processes, interventions, and comparisons; the primary and secondary outcomes to be measured; data management plans; and a detailed description of safety considerations, bias management, and planned statistical analyses.

The study protocol includes the PRISMA-P checklist detailing how each step will be conducted. Funnel plots and Egger’s test will be added if more than 10 studies are included, and the possibility of missing data will be discussed to address publication bias. How the risk of bias is managed using appropriate validated tools, depending on the study design, such as ROBIN 1, PROBAST AI, and ROB 2, is also included in the protocol and all these improve the credibility of the study findings

*‘Disagreements in risk of bias judgments will be resolved by discussion; third-reviewer adjudication will be sought where consensus cannot be reached.’* The plan includes discussion between the two researchers and engagement of a third researcher when agreement is not reached, which is critical for systematic reviews. There is a detailed description of the data analysis, explaining how it will be conducted while accounting for heterogeneity, which is important in the analysis of systematic reviews as described below

*‘Given the anticipated clinical, methodological, and statistical heterogeneity of included studies, results will be synthesized using a structured narrative approach as the primary method, supplemented by quantitative synthesis where feasible.’*

**Discussion**

The study section has highlighted the importance of the results and their potential impact on referral systems in the future. *This review will provide the first systematic synthesis of evidence on the use of AI models to interpret and triage clinical referral documentation across acute and specialist care pathways.* The results will be valuable to health services with high referral volumes and triage processes.

As AI and large language models are increasingly used in health care, education, and system management, this review may clarify their strengths, limitations, and practical value in referral triage. It may also show whether these tools can reduce the burden of labour-intensive tasks and support more efficient care processes.

The findings will need to be interpreted in relation to the limitations of AI use in other settings and to the context in which they are applied. Even so, the review may offer useful guidance for low- and middle-income countries facing workforce and equipment constraints, where effective AI-supported triage could improve efficiency, patient care, and safety.

Overall, the study protocol includes all the relevant components and is well written. I recommend that the study protocol be accepted for publication.
