# Supplementary material for: The use of artificial intelligence models for interpretation and triage of clinical referral letters to acute and specialist care pathways: Protocol for a systematic review
Source: PLoS One. 2026 Aug 6;21(8):e0355476. doi: 10.1371/journal.pone.0355476 (PMC13446580; doi:10.1371/journal.pone.0355476)
Supplement: Supplementary file 4 [file pone.0355476.s005.docx]

Reviewers' comments:

Reviewer's Responses to Questions

**Comments to the Author**

1. Does the manuscript provide a valid rationale for the proposed study, with clearly identified and justified research questions?

The research question outlined is expected to address a valid academic problem or topic and contribute to the base of knowledge in the field.

Reviewer #1: Yes

Reviewer #2: Yes

No changes necessary.

2. Is the protocol technically sound and planned in a manner that will lead to a meaningful outcome and allow testing the stated hypotheses?

The manuscript should describe the methods in sufficient detail to prevent undisclosed flexibility in the experimental procedure or analysis pipeline, including sufficient outcome-neutral conditions (e.g. necessary controls, absence of floor or ceiling effects) to test the proposed hypotheses and a statistical power analysis where applicable. As there may be aspects of the methodology and analysis which can only be refined once the work is undertaken, authors should outline potential assumptions and explicitly describe what aspects of the proposed analyses, if any, are exploratory.

Reviewer #1: Yes

Reviewer #2: Yes

No changes necessary.

3. Is the methodology feasible and described in sufficient detail to allow the work to be replicable?

Descriptions of methods and materials in the protocol should be reported in sufficient detail for another researcher to reproduce all experiments and analyses. The protocol should describe the appropriate controls, sample size calculations, and replication needed to ensure that the data are robust and reproducible.

Reviewer #1: Yes

Reviewer #2: Yes

No changes necessary.

4. Have the authors described where all data underlying the findings will be made available when the study is complete?

The [PLOS Data policy](https://track.editorialmanager.com/CL0/https:%2F%2Fjournals.plos.org%2Fplosone%2Fs%2Fmaterials-and-software-sharing/1/010f019eddfcce9d-667f331b-458a-48f8-a495-80c8fd97d3ab-000000/EqvWcXuHg0OBg-4-kSyov2_sJRJA4P0s6R4G5xFqaps=258) requires authors to make all data underlying the findings described in their manuscript fully available without restriction, with rare exception, at the time of publication. The data should be provided as part of the manuscript or its supporting information, or deposited to a public repository. For example, in addition to summary statistics, the data points behind means, medians and variance measures should be available. If there are restrictions on publicly sharing data—e.g. participant privacy or use of data from a third party—those must be specified.

Reviewer #1: Yes

Reviewer #2: Yes

No changes necessary.

5. Is the manuscript presented in an intelligible fashion and written in standard English?

PLOS ONE does not copyedit accepted manuscripts, so the language in submitted articles must be clear, correct, and unambiguous. Any typographical or grammatical errors should be corrected at revision, so please note any specific errors here.

Reviewer #1: Yes

Reviewer #2: Yes

No changes necessary.

6. Review Comments to the Author

Please use the space provided to explain your answers to the questions above and, if applicable, provide comments about issues authors must address before this protocol can be accepted for publication. You may also include additional comments for the author, including concerns about research or publication ethics.

You may also provide optional suggestions and comments to authors that they might find helpful in planning their study.

Reviewer #1: Thank you for the opportunity to review this protocol. I think this is a timely and worthwhile topic. The motivation is strong, and the authors identify a real problem in clinical systems: referral triage is often slow, variable, resource-intensive, and dependent on clinicians interpreting incomplete or heterogeneous referral information. The idea of systematically reviewing how AI models have been used to interpret referral documentation and support triage is therefore important.

Thank you for your comments.

Overall, the protocol is well structured. The authors have registered the review, use appropriate reporting guidance, describe a broad search strategy, and plan duplicate screening, duplicate extraction, risk-of-bias assessment, and structured narrative synthesis. I also appreciate that the authors already recognise that the included studies are likely to be heterogeneous and that meta-analysis may only be possible in limited circumstances.

Thank you for your comments.

My main concern is not that the methods are poorly designed. Rather, my concern is about the nature of the target being reviewed.
Triage is not a single diagnostic test. It is not quite the same as asking whether a patient has or does not have a disease. Triage is a clinical prioritisation judgement made within a particular pathway, by particular people, using particular information, under particular resource constraints. What counts as “accurate” triage therefore depends heavily on the comparator, the local pathway, the urgency categories, the acceptable balance between over-triage and under-triage, and the clinical consequences of getting the decision wrong. For that reason, I worry that “triage accuracy” may be a moving target across the literature. One study may define accuracy as agreement with an expert clinician. Another may define it as prediction of an urgency category. Another may use waiting-list priority, guideline-based appropriateness, downstream admission, deterioration, or time to review. These may all be reasonable outcomes, but they are not necessarily measuring the same thing. This matters because it may make a conventional diagnostic accuracy meta-analysis difficult to interpret, even if several studies report superficially similar metrics such as accuracy, sensitivity, specificity, or AUC. The same numerical metric may mean quite different things depending on the clinical context and reference standard. I therefore think the authors should consider strengthening the conceptual framing of the protocol. The review may ultimately be most valuable not as a meta-analysis of “AI triage accuracy,” but as a structured synthesis of how this emerging field is defining and evaluating AI-assisted referral triage. In other words, the key contribution may be to map the evidence base: what kinds of AI models have been used, what types of referral documentation they process, what clinical pathways they are applied to, what reference standards are used, how safety is measured, whether calibration and external validation are reported, and how implementation outcomes are handled. This does not mean the review should not be systematic. On the contrary, the systematic approach is valuable. But I would encourage the authors to be cautious about presenting meta-analysis as an expected or central output. A narrative synthesis, or even a scoping-review style evidence map within the systematic review framework, may be the more clinically meaningful product given the heterogeneity of the field.

I would also suggest that the authors consider whether terms such as “clinical acuity grading,” “triage appropriateness,” “prioritisation performance,” or “agreement with a defined clinical reference standard” may be more precise than “diagnostic accuracy” in some parts of the protocol. If the term “accuracy” is retained, it would be helpful to specify that accuracy will always be interpreted in relation to the study’s own reference standard and clinical context, rather than treated as a directly transferable construct.

A related point is that under-triage and over-triage are very important safety outcomes, but their meaning will vary across settings. Under-triage in an emergency referral pathway is not necessarily the same as under-prioritisation in an outpatient specialist waiting list. The protocol would be strengthened by a clearer plan for how these outcomes will be grouped, compared, or narratively interpreted across different triage systems.

Thank you for your helpful comments. We agree and have reframed the review around a dual aim of evidence mapping and performance synthesis. We replaced "diagnostic accuracy" with "prioritisation performance" (defined as agreement with each study's own reference standard), added a secondary objective on mapping the evidence base, and revised the Data synthesis section to make narrative synthesis and evidence mapping the primary output with meta-analysis now conditional and exploratory. We have made edits regarding over and under triage and defined how they might differ according to clinical pathway. Corresponding edits were made to the Abstract and Discussion. The systematic methodology is retained.

Similarly, “AI model” is a very broad category. Traditional machine-learning classifiers, NLP pipelines, deep learning models, and LLM-based systems have very different validation requirements and different failure modes. The protocol already plans to extract model type and architecture, which is good, but I would encourage the authors to make this stratification central to the synthesis. It may be more informative to compare how different model classes have been evaluated than to try to combine them under a single umbrella of AI performance.

We agree. Stratification by AI model class (traditional ML classifiers, NLP pipelines, deep learning, and LLM-based systems) is now a central organising principle of the synthesis, extending beyond performance to how each class is evaluated; validation designs, reference standards, and reported failure modes. We have added model class and reported failure modes to the data extraction form to support this.

There are also some smaller methodological points that I think would improve the protocol:

First, the authors should clarify the threshold for attempting meta-analysis. The statement that meta-analysis will be performed where at least three studies report comparable outcomes is reasonable as a minimum, but comparability should include more than the metric reported. It should include clinical setting, reference standard, outcome definition, and threshold structure.

Thanks, this has been clarified in the text, and edits define appropriately.

Second, if diagnostic accuracy meta-analysis is performed, the authors should specify methods appropriate for diagnostic accuracy data, such as bivariate random-effects or HSROC models, particularly where sensitivity and specificity are being synthesised and thresholds differ between studies.

Thank you for this suggestion. This has been specified in the Data synthesis section: where diagnostic accuracy meta-analysis is appropriate, bivariate random-effects or hierarchical summary receiver operating characteristic (HSROC) models will be used, depending on the availability of sensitivity and specificity estimates and the degree of threshold variation across studies.

Third, the risk-of-bias approach could be clarified. PROBAST-AI is appropriate for prediction model studies, and RoB 2/ROBINS-I are appropriate for comparative intervention studies. However, if studies are being treated as diagnostic accuracy studies, the authors may wish to consider QUADAS-2 or explain why PROBAST-AI is the preferred tool for those designs.

Thank you for your suggestion. QUADAS-2 has been considered, and added as per your suggestion, with reference.

Fourth, given how fast the AI literature is moving, the authors may wish to explicitly consider preprints, citation chaining, and possibly trial or model registries. This does not mean preprints should be treated as equivalent to peer-reviewed studies, but a clear plan for identifying and handling them would improve transparency.

Thank you for this suggestion. We have added forward and backward citation chaining of all included studies and relevant reviews, and a search of Europe PMC for relevant preprints. Preprints will be screened against the same eligibility criteria, flagged as non-peer-reviewed, and reported separately; they will not be pooled with peer-reviewed studies in any quantitative synthesis (Information sources and search strategy). We considered trial and model registries but elected not to search them, as the review is restricted to studies reporting quantitative evaluation of AI performance, which registry entries alone do not provide; citation chaining and preprint searching were prioritised as the higher-yield supplementary sources for this literature.

Fifth, the protocol should include a plan for overlapping datasets, multiple publications from the same model or cohort, and evolving model versions. This is particularly relevant in AI research, where the same dataset or model family may appear across several papers.

We have added a plan to the Selection process. Where multiple publications report on the same model, dataset, or cohort, the most complete and rigorous will be designated the primary record to avoid double-counting, and successive model versions (e.g. GPT-3.5 vs GPT-4) will be reported separately with version, release date, and fine-tuning extracted.

Finally, there appear to be minor issues that should be corrected, including the inconsistent PROSPERO registration number and clarification of the statistical software version.

Both have been corrected. The PROSPERO registration number now reads CRD420251244654 consistently throughout the manuscript, and the statistical software is now uniformly cited as StataNow 19.5 SE (StataCorp LLC, 2025) in both the text and the reference list.

In summary, I think this is a useful and publishable protocol. The topic is important, the authors have approached it thoughtfully, and the methods are broadly appropriate. My recommendation is that the authors revise the protocol to make the central conceptual issue clearer: triage is a context-dependent clinical prioritisation process, not a single stable diagnostic target. If this is acknowledged more explicitly, and if meta-analysis is framed as conditional and exploratory rather than expected, the eventual review is likely to be more clinically meaningful and more useful to readers.

Thank you for your comments.

Reviewer #2: The use of artificial intelligence models for interpretation and triage of clinical referral letters to acute and specialist care pathways: protocol for a systematic review. This topic, titled above, is quite relevant and important in primary health care, as it affects patients' waiting times to be seen at specialist referral centers and/or for other acute clinical care. The sections are well written and in accordance with the journal's published guidelines and requirements for publishing the study protocol, as required by PLOS ONE. Each heading is accompanied by an adequate description that clarifies the steps required for the study protocol submission guidelines. The study protocol has implications; hence, if published, the detailed protocol can be used to review the findings and advantages, enabling replication of the research in other contexts when relevant. ‘Systematic reviews aim to provide a comprehensive, unbiased synthesis of many relevant studies in a single document using rigorous and transparent methods’ which is achieved through the study protocol.

Introduction
‘This section is well written and makes a case for introducing different models, such as AI, to tackle a common problem in the referral system. This can fast-track patient triage, enabling them to be attended to according to their priority and providing quicker, more appropriate, and optimal care. Each topic is well developed, with a clear paragraph structure and creates an argument. The aim and objectives in this study protocol are clearly stated.’

Thank you for your comments.

Study Materials and Methods
The guidelines require that they should include the aim, design, and setting of the study, the sample size, inclusion, and exclusion criteria, the characteristics of participants and how the sample will be selected, and, where applicable, randomization and blinding and description of how materials will be selected for the study. A true description of all processes, interventions, comparisons, what outcomes will be measured as primary and secondary outcomes, data management plans and a detailed description of safety considerations, bias management, and statistical analyses planned are all explained in the study protocol. In this study protocol, the researchers clearly describe how the planned systematic review will be conducted using rigorous and transparent methods. The guidelines also require a clear explanation of the study aim, design, and setting; sample size; inclusion and exclusion criteria; participant characteristics; sample selection procedures; and, where applicable, randomization, blinding, and the process used to select studies. These components are well explained in the protocol. All of the following are explicitly detailed in the study protocol: a true description of all processes, interventions, and comparisons; the primary and secondary outcomes to be measured; data management plans; and a detailed description of safety considerations, bias management, and planned statistical analyses.

Thank you for your comments.

The study protocol includes the PRISMA-P checklist detailing how each step will be conducted. Funnel plots and Egger’s test will be added if more than 10 studies are included, and the possibility of missing data will be discussed to address publication bias. How the risk of bias is managed using appropriate validated tools, depending on the study design, such as ROBIN 1, PROBAST AI, and ROB 2, is also included in the protocol and all these improve the credibility of the study findings.

Thank you for your comments.

‘Disagreements in risk of bias judgments will be resolved by discussion; third-reviewer adjudication will be sought where consensus cannot be reached.’ The plan includes discussion between the two researchers and engagement of a third researcher when agreement is not reached, which is critical for systematic reviews. There is a detailed description of the data analysis, explaining how it will be conducted while accounting for heterogeneity, which is important in the analysis of systematic reviews as described below
‘Given the anticipated clinical, methodological, and statistical heterogeneity of included studies, results will be synthesized using a structured narrative approach as the primary method, supplemented by quantitative synthesis where feasible.’

Thank you for your comments.

Discussion
The study section has highlighted the importance of the results and their potential impact on referral systems in the future. This review will provide the first systematic synthesis of evidence on the use of AI models to interpret and triage clinical referral documentation across acute and specialist care pathways. The results will be valuable to health services with high referral volumes and triage processes.

As AI and large language models are increasingly used in health care, education, and system management, this review may clarify their strengths, limitations, and practical value in referral triage. It may also show whether these tools can reduce the burden of labour-intensive tasks and support more efficient care processes.

The findings will need to be interpreted in relation to the limitations of AI use in other settings and to the context in which they are applied. Even so, the review may offer useful guidance for low- and middle-income countries facing workforce and equipment constraints, where effective AI-supported triage could improve efficiency, patient care, and safety.

Overall, the study protocol includes all the relevant components and is well written. I recommend that the study protocol be accepted for publication.

Thank you for your comments.
