# Supplementary material for: The use of artificial intelligence models for interpretation and triage of clinical referral letters to acute and specialist care pathways: Protocol for a systematic review
Source: PLoS One. 2026 Aug 6;21(8):e0355476. doi: 10.1371/journal.pone.0355476 (PMC13446580; doi:10.1371/journal.pone.0355476)
Supplement: Peer review history

# Peer review: Decision Letter 0

**DOI:** 10.1371/journal.pone.0355476.r001

**Authors:** Mergan Naidoo

18 Jun 2026

PONE-D-26-21020

The use of artificial intelligence models for interpretation and triage of clinical referral letters to acute and specialist care pathways: protocol for a systematic review

PLOS One

Dear Dr. Foley,

Thank you for submitting your manuscript to PLOS ONE. After careful consideration, we feel that it has merit but does not fully meet PLOS ONE’s publication criteria as it currently stands. The reviewers have suggested minor changes to the protocol. Therefore, we invite you to submit a revised version of the manuscript that addresses the points raised during the review process.

Please submit your revised manuscript by Aug 02 2026 11:59PM. If you will need more time than this to complete your revisions, please reply to this message or contact the journal office at plosone@plos.org. When you're ready to submit your revision, log on to https://www.editorialmanager.com/pone/ and select the 'Submissions Needing Revision' folder to locate your manuscript file.

Please include the following items when submitting your revised manuscript:

- A letter that responds to each point raised by the academic editor and reviewer(s). You should upload this letter as a separate file labeled 'Response to Reviewers'.
- A marked-up copy of your manuscript that highlights changes made to the original version. You should upload this as a separate file labeled 'Revised Manuscript with Track Changes'.
- An unmarked version of your revised paper without tracked changes. You should upload this as a separate file labeled 'Manuscript'.

If you would like to make changes to your financial disclosure, please include your updated statement in your cover letter. Guidelines for resubmitting your figure files are available below the reviewer comments at the end of this letter.

If applicable, we recommend that you deposit your laboratory protocols in protocols.io to enhance the reproducibility of your results. Protocols.io assigns your protocol its own identifier (DOI) so that it can be cited independently in the future. For instructions see: https://journals.plos.org/plosone/s/submission-guidelines#loc-laboratory-protocols. Additionally, PLOS ONE offers an option for publishing peer-reviewed Lab Protocol articles, which describe protocols hosted on protocols.io. Read more information on sharing protocols at https://plos.org/protocols?utm_medium=editorial-email&utm_source=authorletters&utm_campaign=protocols.

As the corresponding author, your ORCID iD is verified in the submission system and will appear in the published article. PLOS supports the use of ORCID, and we encourage all coauthors to register for an ORCID iD and use it as well. Please encourage your coauthors to verify their ORCID iD within the submission system before final acceptance, as unverified ORCID iDs will not appear in the published article. *Only*  the individual author can complete the verification step; PLOS staff *cannot*  verify ORCID iDs on behalf of authors.

We look forward to receiving your revised manuscript.

Kind regards,

Mergan Naidoo, PhD

Academic Editor

PLOS One

Journal Requirements:

When submitting your revision, we need you to address these additional requirements.

1. Please ensure that your manuscript meets PLOS ONE's style requirements, including those for file naming. The PLOS ONE style templates can be found at

https://journals.plos.org/plosone/s/file?id=wjVg/PLOSOne_formatting_sample_main_body.pdf and

https://journals.plos.org/plosone/s/file?id=ba62/PLOSOne_formatting_sample_title_authors_affiliations.pdf

2. Thank you for stating the following in your Competing Interests section: [None declared.].

Please complete your Competing Interests on the online submission form to state any Competing Interests. If you have no competing interests, please state ""The authors have declared that no competing interests exist."", as detailed online in our guide for authors at http://journals.plos.org/plosone/s/submit-now

This information should be included in your cover letter; we will change the online submission form on your behalf.

3. Please include captions for your Supporting Information files at the end of your manuscript, and update any in-text citations to match accordingly. Please see our Supporting Information guidelines for more information: http://journals.plos.org/plosone/s/supporting-information.

4. If the reviewer comments include a recommendation to cite specific previously published works, please review and evaluate these publications to determine whether they are relevant and should be cited. There is no requirement to cite these works unless the editor has indicated otherwise.

5. Please review your reference list to ensure that it is complete and correct. If you have cited papers that have been retracted, please include the rationale for doing so in the manuscript text, or remove these references and replace them with relevant current references. Any changes to the reference list should be mentioned in the rebuttal letter that accompanies your revised manuscript. If you need to cite a retracted article, indicate the article’s retracted status in the References list and also include a citation and full reference for the retraction notice.

[Note: HTML markup is below. Please do not edit.]

Reviewers' comments:

Reviewer's Responses to Questions

**Comments to the Author**

1. Does the manuscript provide a valid rationale for the proposed study, with clearly identified and justified research questions?

The research question outlined is expected to address a valid academic problem or topic and contribute to the base of knowledge in the field.

Reviewer #1: Yes

Reviewer #2: Yes

**********

2. Is the protocol technically sound and planned in a manner that will lead to a meaningful outcome and allow testing the stated hypotheses?

The manuscript should describe the methods in sufficient detail to prevent undisclosed flexibility in the experimental procedure or analysis pipeline, including sufficient outcome-neutral conditions (e.g. necessary controls, absence of floor or ceiling effects) to test the proposed hypotheses and a statistical power analysis where applicable. As there may be aspects of the methodology and analysis which can only be refined once the work is undertaken, authors should outline potential assumptions and explicitly describe what aspects of the proposed analyses, if any, are exploratory.

Reviewer #1: Yes

Reviewer #2: Yes

**********

3. Is the methodology feasible and described in sufficient detail to allow the work to be replicable?

Descriptions of methods and materials in the protocol should be reported in sufficient detail for another researcher to reproduce all experiments and analyses. The protocol should describe the appropriate controls, sample size calculations, and replication needed to ensure that the data are robust and reproducible.

Reviewer #1: Yes

Reviewer #2: Yes

**********

4. Have the authors described where all data underlying the findings will be made available when the study is complete?

The PLOS Data policy requires authors to make all data underlying the findings described in their manuscript fully available without restriction, with rare exception, at the time of publication. The data should be provided as part of the manuscript or its supporting information, or deposited to a public repository. For example, in addition to summary statistics, the data points behind means, medians and variance measures should be available. If there are restrictions on publicly sharing data—e.g. participant privacy or use of data from a third party—those must be specified.

Reviewer #1: Yes

Reviewer #2: Yes

**********

5. Is the manuscript presented in an intelligible fashion and written in standard English?

PLOS ONE does not copyedit accepted manuscripts, so the language in submitted articles must be clear, correct, and unambiguous. Any typographical or grammatical errors should be corrected at revision, so please note any specific errors here.

Reviewer #1: Yes

Reviewer #2: Yes

**********

6. Review Comments to the Author

Please use the space provided to explain your answers to the questions above and, if applicable, provide comments about issues authors must address before this protocol can be accepted for publication. You may also include additional comments for the author, including concerns about research or publication ethics.

You may also provide optional suggestions and comments to authors that they might find helpful in planning their study.

(Please upload your review as an attachment if it exceeds 20,000 characters)

Reviewer #1: Thank you for the opportunity to review this protocol. I think this is a timely and worthwhile topic. The motivation is strong, and the authors identify a real problem in clinical systems: referral triage is often slow, variable, resource-intensive, and dependent on clinicians interpreting incomplete or heterogeneous referral information. The idea of systematically reviewing how AI models have been used to interpret referral documentation and support triage is therefore important.

Overall, the protocol is well structured. The authors have registered the review, use appropriate reporting guidance, describe a broad search strategy, and plan duplicate screening, duplicate extraction, risk-of-bias assessment, and structured narrative synthesis. I also appreciate that the authors already recognise that the included studies are likely to be heterogeneous and that meta-analysis may only be possible in limited circumstances.

My main concern is not that the methods are poorly designed. Rather, my concern is about the nature of the target being reviewed.

Triage is not a single diagnostic test. It is not quite the same as asking whether a patient has or does not have a disease. Triage is a clinical prioritisation judgement made within a particular pathway, by particular people, using particular information, under particular resource constraints. What counts as “accurate” triage therefore depends heavily on the comparator, the local pathway, the urgency categories, the acceptable balance between over-triage and under-triage, and the clinical consequences of getting the decision wrong.

For that reason, I worry that “triage accuracy” may be a moving target across the literature. One study may define accuracy as agreement with an expert clinician. Another may define it as prediction of an urgency category. Another may use waiting-list priority, guideline-based appropriateness, downstream admission, deterioration, or time to review. These may all be reasonable outcomes, but they are not necessarily measuring the same thing.

This matters because it may make a conventional diagnostic accuracy meta-analysis difficult to interpret, even if several studies report superficially similar metrics such as accuracy, sensitivity, specificity, or AUC. The same numerical metric may mean quite different things depending on the clinical context and reference standard.

I therefore think the authors should consider strengthening the conceptual framing of the protocol. The review may ultimately be most valuable not as a meta-analysis of “AI triage accuracy,” but as a structured synthesis of how this emerging field is defining and evaluating AI-assisted referral triage. In other words, the key contribution may be to map the evidence base: what kinds of AI models have been used, what types of referral documentation they process, what clinical pathways they are applied to, what reference standards are used, how safety is measured, whether calibration and external validation are reported, and how implementation outcomes are handled.

This does not mean the review should not be systematic. On the contrary, the systematic approach is valuable. But I would encourage the authors to be cautious about presenting meta-analysis as an expected or central output. A narrative synthesis, or even a scoping-review style evidence map within the systematic review framework, may be the more clinically meaningful product given the heterogeneity of the field.

I would also suggest that the authors consider whether terms such as “clinical acuity grading,” “triage appropriateness,” “prioritisation performance,” or “agreement with a defined clinical reference standard” may be more precise than “diagnostic accuracy” in some parts of the protocol. If the term “accuracy” is retained, it would be helpful to specify that accuracy will always be interpreted in relation to the study’s own reference standard and clinical context, rather than treated as a directly transferable construct.

A related point is that under-triage and over-triage are very important safety outcomes, but their meaning will vary across settings. Under-triage in an emergency referral pathway is not necessarily the same as under-prioritisation in an outpatient specialist waiting list. The protocol would be strengthened by a clearer plan for how these outcomes will be grouped, compared, or narratively interpreted across different triage systems.

Similarly, “AI model” is a very broad category. Traditional machine-learning classifiers, NLP pipelines, deep learning models, and LLM-based systems have very different validation requirements and different failure modes. The protocol already plans to extract model type and architecture, which is good, but I would encourage the authors to make this stratification central to the synthesis. It may be more informative to compare how different model classes have been evaluated than to try to combine them under a single umbrella of AI performance.

There are also some smaller methodological points that I think would improve the protocol:

First, the authors should clarify the threshold for attempting meta-analysis. The statement that meta-analysis will be performed where at least three studies report comparable outcomes is reasonable as a minimum, but comparability should include more than the metric reported. It should include clinical setting, reference standard, outcome definition, and threshold structure.

Second, if diagnostic accuracy meta-analysis is performed, the authors should specify methods appropriate for diagnostic accuracy data, such as bivariate random-effects or HSROC models, particularly where sensitivity and specificity are being synthesised and thresholds differ between studies.

Third, the risk-of-bias approach could be clarified. PROBAST-AI is appropriate for prediction model studies, and RoB 2/ROBINS-I are appropriate for comparative intervention studies. However, if studies are being treated as diagnostic accuracy studies, the authors may wish to consider QUADAS-2 or explain why PROBAST-AI is the preferred tool for those designs.

Fourth, given how fast the AI literature is moving, the authors may wish to explicitly consider preprints, citation chaining, and possibly trial or model registries. This does not mean preprints should be treated as equivalent to peer-reviewed studies, but a clear plan for identifying and handling them would improve transparency.

Fifth, the protocol should include a plan for overlapping datasets, multiple publications from the same model or cohort, and evolving model versions. This is particularly relevant in AI research, where the same dataset or model family may appear across several papers.

Finally, there appear to be minor issues that should be corrected, including the inconsistent PROSPERO registration number and clarification of the statistical software version.

In summary, I think this is a useful and publishable protocol. The topic is important, the authors have approached it thoughtfully, and the methods are broadly appropriate. My recommendation is that the authors revise the protocol to make the central conceptual issue clearer: triage is a context-dependent clinical prioritisation process, not a single stable diagnostic target. If this is acknowledged more explicitly, and if meta-analysis is framed as conditional and exploratory rather than expected, the eventual review is likely to be more clinically meaningful and more useful to readers.

Reviewer #2: The use of artificial intelligence models for interpretation and triage of clinical referral letters to acute and specialist care pathways: protocol for a systematic review

This topic, titled above, is quite relevant and important in primary health care, as it affects patients' waiting times to be seen at specialist referral centers and/or for other acute clinical care.

The sections are well written and in accordance with the journal's published guidelines and requirements for publishing the study protocol, as required by PLOS ONE. Each heading is accompanied by an adequate description that clarifies the steps required for the study protocol submission guidelines.

The study protocol has implications; hence, if published, the detailed protocol can be used to review the findings and advantages, enabling replication of the research in other contexts when relevant.

‘Systematic reviews aim to provide a comprehensive, unbiased synthesis of many relevant studies in a single document using rigorous and transparent methods’ which is achieved through the study protocol

Introduction

‘This section is well written and makes a case for introducing different models, such as AI, to tackle a common problem in the referral system. This can fast-track patient triage, enabling them to be attended to according to their priority and providing quicker, more appropriate, and optimal care. Each topic is well developed, with a clear paragraph structure and creates an argument. The aim and objectives in this study protocol are clearly stated.’

Study Materials and Methods

The guidelines require that they should include the aim, design, and setting of the study, the sample size, inclusion, and exclusion criteria, the characteristics of participants and how the sample will be selected, and, where applicable, randomization and blinding and description of how materials will be selected for the study

A true description of all processes, interventions, comparisons, what outcomes will be measured as primary and secondary outcomes, data management plans and a detailed description of safety considerations, bias management, and statistical analyses planned are all explained in the study protocol

In this study protocol, the researchers clearly describe how the planned systematic review will be conducted using rigorous and transparent methods.

The guidelines also require a clear explanation of the study aim, design, and setting; sample size; inclusion and exclusion criteria; participant characteristics; sample selection procedures; and, where applicable, randomization, blinding, and the process used to select studies. These components are well explained in the protocol.

All of the following are explicitly detailed in the study protocol: a true description of all processes, interventions, and comparisons; the primary and secondary outcomes to be measured; data management plans; and a detailed description of safety considerations, bias management, and planned statistical analyses.

The study protocol includes the PRISMA-P checklist detailing how each step will be conducted. Funnel plots and Egger’s test will be added if more than 10 studies are included, and the possibility of missing data will be discussed to address publication bias. How the risk of bias is managed using appropriate validated tools, depending on the study design, such as ROBIN 1, PROBAST AI, and ROB 2, is also included in the protocol and all these improve the credibility of the study findings

‘Disagreements in risk of bias judgments will be resolved by discussion; third-reviewer adjudication will be sought where consensus cannot be reached.’ The plan includes discussion between the two researchers and engagement of a third researcher when agreement is not reached, which is critical for systematic reviews. There is a detailed description of the data analysis, explaining how it will be conducted while accounting for heterogeneity, which is important in the analysis of systematic reviews as described below

‘Given the anticipated clinical, methodological, and statistical heterogeneity of included studies, results will be synthesized using a structured narrative approach as the primary method, supplemented by quantitative synthesis where feasible.’

Discussion

The study section has highlighted the importance of the results and their potential impact on referral systems in the future. This review will provide the first systematic synthesis of evidence on the use of AI models to interpret and triage clinical referral documentation across acute and specialist care pathways. The results will be valuable to health services with high referral volumes and triage processes.

As AI and large language models are increasingly used in health care, education, and system management, this review may clarify their strengths, limitations, and practical value in referral triage. It may also show whether these tools can reduce the burden of labour-intensive tasks and support more efficient care processes.

The findings will need to be interpreted in relation to the limitations of AI use in other settings and to the context in which they are applied. Even so, the review may offer useful guidance for low- and middle-income countries facing workforce and equipment constraints, where effective AI-supported triage could improve efficiency, patient care, and safety.

Overall, the study protocol includes all the relevant components and is well written. I recommend that the study protocol be accepted for publication.

**********

7. PLOS authors have the option to publish the peer review history of their article (what does this mean?). If published, this will include your full peer review and any attached files.

If you choose “no”, your identity will remain anonymous but your review may still be made public.

**Do you want your identity to be public for this peer review?**  For information about this choice, including consent withdrawal, please see our Privacy Policy.

Reviewer #1: **Yes:** Kamlin Ekambaram

Reviewer #2: No

**********

[NOTE: If reviewer comments were submitted as an attachment file, they will be attached to this email and accessible via the submission site. Please log into your account, locate the manuscript record, and check for the action link "View Attachments". If this link does not appear, there are no attachment files.]

To ensure your figures meet our technical requirements, please review our figure guidelines: https://journals.plos.org/plosone/s/figures

You may also use PLOS’s free figure tool, NAAS, to help you prepare publication quality figures: https://journals.plos.org/plosone/s/figures#loc-tools-for-figure-preparation.

NAAS will assess whether your figures meet our technical requirements by comparing each figure against our figure specifications.

Attachment Submitted filename: REVIEWER COMMENTS F.docx

# Author comment: Author response to Decision Letter 1

**DOI:** 10.1371/journal.pone.0355476.r002

2 Jul 2026

Please see the attached response to reviewers document.

Attachment Submitted filename: Response to Reviewers JF 220626.docx

# Editor report: Decision Letter 1

**DOI:** 10.1371/journal.pone.0355476.r003

**Authors:** Mergan Naidoo

22 Jul 2026

The use of artificial intelligence models for interpretation and triage of clinical referral letters to acute and specialist care pathways: protocol for a systematic review

PONE-D-26-21020R1

Dear Dr. James Foley

We’re pleased to inform you that your manuscript has been judged scientifically suitable for publication and will be formally accepted for publication once it meets all outstanding technical requirements.

Within one week, you’ll receive an e-mail detailing the required amendments. When these have been addressed, you’ll receive a formal acceptance letter and your manuscript will be scheduled for publication.

An invoice will be generated when your article is formally accepted. Please note, if your institution has a publishing partnership with PLOS and your article meets the relevant criteria, all or part of your publication costs will be covered. Please make sure your user information is up-to-date by logging into Editorial Manager at Editorial Manager® and clicking the ‘Update My Information' link at the top of the page. For questions related to billing, please contact billing support.

If your institution or institutions have a press office, please notify them about your upcoming paper to help maximize its impact. If they’ll be preparing press materials, please inform our press team as soon as possible -- no later than 48 hours after receiving the formal acceptance. Your manuscript will remain under strict press embargo until 2 pm Eastern Time on the date of publication. For more information, please contact onepress@plos.org.

Kind regards,

Mergan Naidoo, PhD

Academic Editor

PLOS One

Additional Editor Comments (optional):

Reviewers' comments:

# Editor report: Acceptance letter

**DOI:** 10.1371/journal.pone.0355476.r004

**Authors:** Mergan Naidoo

PONE-D-26-21020R1

PLOS One

Dear Dr. Foley,

I'm pleased to inform you that your manuscript has been deemed suitable for publication in PLOS One. Congratulations! Your manuscript is now being handed over to our production team.

At this stage, our production department will prepare your paper for publication. This includes ensuring the following:

* All references, tables, and figures are properly cited

* All relevant supporting information is included in the manuscript submission,

* There are no issues that prevent the paper from being properly typeset

You will receive further instructions from the production team, including instructions on how to review your proof when it is ready. Please keep in mind that we are working through a large volume of accepted articles, so please give us a few days to review your paper and let you know the next and final steps.

Lastly, if your institution or institutions have a press office, please let them know about your upcoming paper now to help maximize its impact. If they'll be preparing press materials, please inform our press team within the next 48 hours. Your manuscript will remain under strict press embargo until 2 pm Eastern Time on the date of publication. For more information, please contact onepress@plos.org.

You will receive an invoice from PLOS for your publication fee after your manuscript has reached the completed accept phase. If you receive an email requesting payment before acceptance or for any other service, this may be a phishing scheme. Learn how to identify phishing emails and protect your accounts at https://explore.plos.org/phishing.

If we can help with anything else, please email us at customercare@plos.org.

Thank you for submitting your work to PLOS ONE and supporting open access.

Kind regards,

PLOS ONE Editorial Office Staff

on behalf of

Professor Mergan Naidoo

Academic Editor

PLOS One
